# Genome Sequences of Two Soil-Dwelling Bacteria Belonging to the Family *Comamonadaceae*

**DOI:** 10.1128/mra.00230-22

**Published:** 2022-08-23

**Authors:** Adrien Biessy, Marie Ciotola, Mélanie Cadieux, Daphné Albert, Martin Filion

**Affiliations:** a Saint-Jean-sur-Richelieu Research and Development Centre, Agriculture and Agri-Food Canada, Saint-Jean-sur-Richelieu, Quebec, Canada; SIPBS, University of Strathclyde

## Abstract

Members of the family *Comamonadaceae* are rod-shaped betaproteobacteria found in various environments. Here, we report the genome sequences of 2 bacteria belonging to this family. They were isolated from agricultural soils located in the Montérégie region (Quebec, Canada) and display biocontrol activity against various lettuce bacterial pathogens.

## ANNOUNCEMENT

Two bacterial strains belonging to the family *Comamonadaceae* were isolated in 2019 from agricultural soils located in the Montérégie region (Quebec, Canada). These 2 strains were shown to display biocontrol activity toward several lettuce bacterial pathogens. They were isolated as previously described (1). Briefly, soil samples were collected as close as possible to the plant roots of various vegetable crop species grown in two agricultural fields located in Dunham and Brigham (Quebec, Canada). Samples were stored at 4°C. One gram of soil was added to 100 mL of saline solution (0.9% NaCl), and the suspension was agitated for 10 min at 250 rpm. The suspension was serially diluted and plated on King’s B agar (2) supplemented with cycloheximide (100 μg mL^−1^), ampicillin (40 μg mL^−1^), and chloramphenicol (13 μg mL^−1^). The plates were incubated at 25°C for 48 h. Isolated colonies were subsequently purified on King’s B agar (25°C for 48 h). The bacterial strains were kept at −80°C in tryptic soy broth (BD Biosciences) supplemented with 10% glycerol (vol/vol). The bacteria were grown in King’s B agar for 48h at 25°C and genomic DNA was extracted with the DNeasy UltraClean microbial kit (Qiagen) according to the manufacturer’s instructions. Genomic DNA was mechanically sheared to obtain 9 to 10 kb fragments using Covaris g-TUBEs (Covaris). Libraries were prepared using the PacBio SMRTbell Express template prep kit (Pacific Biosciences) and the genomes were sequenced on a PacBio Sequel sequencer (v3 chemistry) at the Integrated Microbiome Resource (Halifax, NS, Canada). The quality of the raw reads was checked with FastQC v0.11.9 (3). Genome assembly was performed using the long-read assembler Flye v2.8.1 (4). Default parameters were used for all software unless otherwise specified. The genome of B21-011 was assembled in three contigs, while the genome of B21-038 was assembled in one contig. The 2 genomes were annotated by the NCBI Prokaryotic Genome Annotation Pipeline v6.0 (5). Genomic features (including genome size, GC % content and number of coding DNA sequences) are presented in Table 1.

**TABLE 1:** Genomic features of the two sequenced strains

Train	B21-011	B21-038
Genome size (Mb)	6.38	5.18
GC content (%)	66.8	63.6
contigs	3	1
Contig N50	4.23	5.18
coverage (x)	94	246
No of reads	111,021	345,657
avg read length	6028	4106
Read N50	9423	6375
No of CDSs^*a*^	5638	4620
No of pseudogenes	33	66
No of rRNAs	14	19
No of tRNAs	80	84
GenBank accession	JAKRZQ000000000	CP092462
SRA accession	SRR18054574	SRR18054573

*^a^* CDSs, coding DNA sequences.

**FIG 1:**
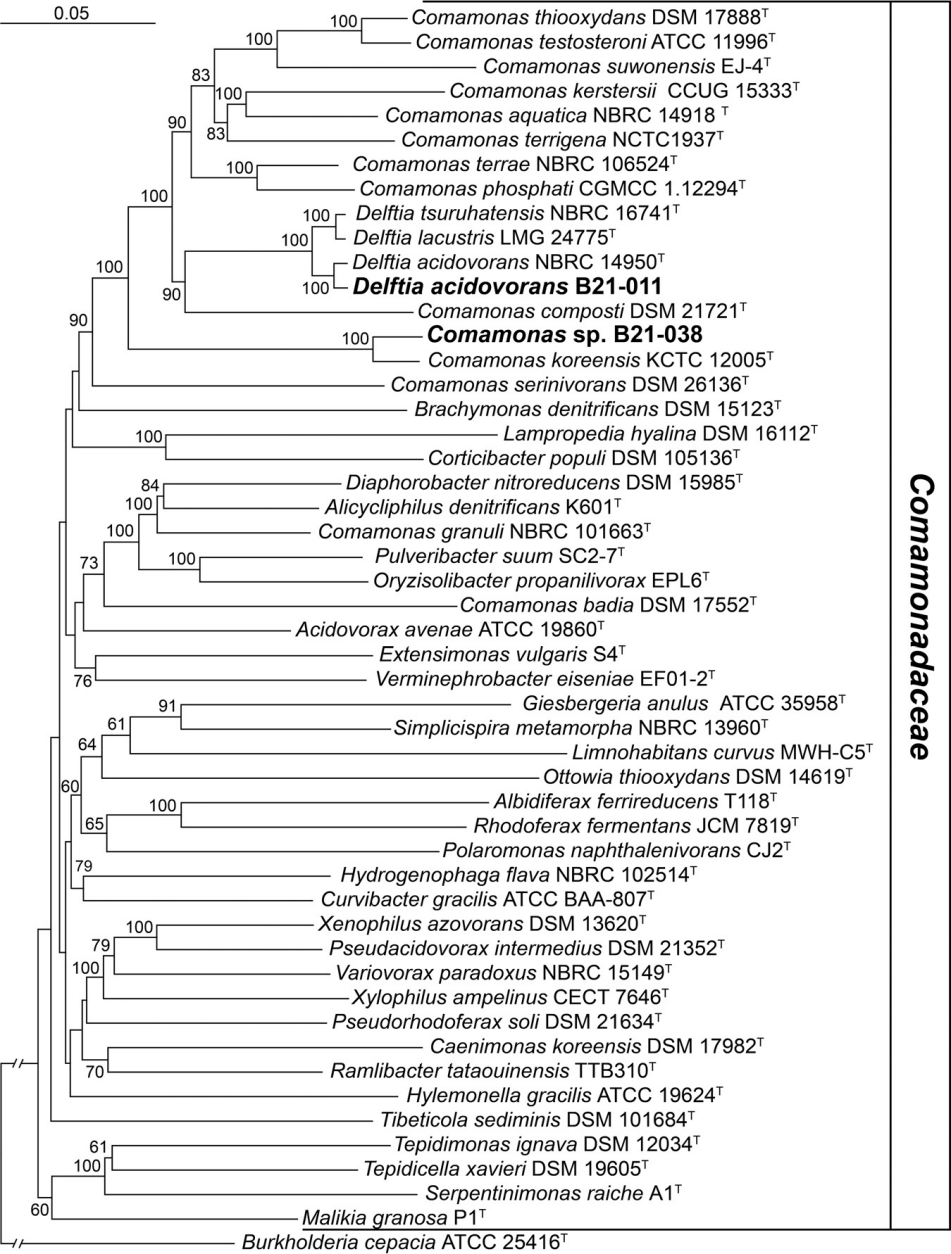
Neighbor-joining phylogeny of the family *Comamonadaceae*. The complete nucleotide sequences of seven housekeeping genes (*atpD*, *gltB*, *gyrB*, *lepA*, *phaC*, *recA*, and *trpB*) were concatenated and subsequently aligned using MUSCLE v3.8.425 (10). The phylogenetic tree was generated from the entire alignment using the Geneious tree builder (Biomatters, Auckland, New Zealand) with the Jukes-Cantor method. Strains whose genomes are reported in this study are highlighted in bold. Only bootstrap values above 60% (from 1000 replicates) are shown. *Bukholderia cepacia* ATCC 25416^T^ was used as an outgroup.

To understand the phylogenetic relationship between these 2 strains and the different species and genera within the family *Comamonadaceae*, we performed a multilocus sequence analysis using 7 housekeeping genes (*atpD*, *gltB*, *gyrB*, *lepA*, *phaC*, *recA*, and *trpB*) (Fig. 1). The 2 strains under study clustered with several type strains belonging to the genera *Comamonas* and *Delftia*. Species-level identification of the two sequenced strains was performed using the Type (Strain) Genome Server (TYGS) (6). Digital DNA-DNA hybridization (dDDH) values were obtained from the TYGS on 2022-04-25. B21-011 belongs to the species Delftia acidovorans (dDDH = 83.1% with Delftia acidovorans NBRC 14950^T^). B21-038 does not belong to any validly described species, the closest type strain being Comamonas koreensis KCTC 12005^T^ (dDDH = 50.5%).

We searched for biosynthetic gene clusters (BGCs) that could contribute to pathogen growth inhibition using antiSMASH 6.0 (7). Delftia acidovorans B21-011 harbors a BGC similar (> 95% identity) to the BGC responsible for the production of delftibactin, a metallophore produced by several strains of D. acidovorans (8) and displaying antimicrobial activity (9). Moreover, D. acidovorans B21-011 harbors a putative type VI secretion system gene cluster. We did not find any BGC associated with plant pathogen suppression in the genome of B21-038.

### Data availability.

The complete genomes of strains B21-011 and B21-038 have been deposited at DDBJ/ENA/GenBank under the following accession numbers: JAKRZQ000000000 (B21-011) and CP092462 (B21-038). The raw sequencing data have been deposited into the Sequence Read Archive (BioProject PRJNA806951) under the following accession numbers: SRR18054574 (B21-011) and SRR18054573 (B21-038). The versions described in this paper are the first versions.
